# Genome-Wide and Functional Annotation of Human E3 Ubiquitin Ligases Identifies MULAN, a Mitochondrial E3 that Regulates the Organelle's Dynamics and Signaling

**DOI:** 10.1371/journal.pone.0001487

**Published:** 2008-01-23

**Authors:** Wei Li, Mario H. Bengtson, Axel Ulbrich, Akio Matsuda, Venkateshwar A. Reddy, Anthony Orth, Sumit K. Chanda, Serge Batalov, Claudio A. P. Joazeiro

**Affiliations:** 1 Department of Cell Biology, The Scripps Research Institute, La Jolla, California, United States of America; 2 Institute for Life Science Research, ASAHI KASEI Corporation, Fuji-shi, Shizuoka, Japan; 3 The Genomics Institute of the Novartis Research Foundation, San Diego, California, United States of America; Whitehead Institute, United States of America

## Abstract

Specificity of protein ubiquitylation is conferred by E3 ubiquitin (Ub) ligases. We have annotated ∼617 putative E3s and substrate-recognition subunits of E3 complexes encoded in the human genome. The limited knowledge of the function of members of the large E3 superfamily prompted us to generate genome-wide E3 cDNA and RNAi expression libraries designed for functional screening. An imaging-based screen using these libraries to identify E3s that regulate mitochondrial dynamics uncovered MULAN/FLJ12875, a RING finger protein whose ectopic expression and knockdown both interfered with mitochondrial trafficking and morphology. We found that MULAN is a mitochondrial protein – two transmembrane domains mediate its localization to the organelle's outer membrane. MULAN is oriented such that its E3-active, C-terminal RING finger is exposed to the cytosol, where it has access to other components of the Ub system. Both an intact RING finger and the correct subcellular localization were required for regulation of mitochondrial dynamics, suggesting that MULAN's downstream effectors are proteins that are either integral to, or associated with, mitochondria and that become modified with Ub. Interestingly, MULAN had previously been identified as an activator of NF-κB, thus providing a link between mitochondrial dynamics and mitochondria-to-nucleus signaling. These findings suggest the existence of a new, Ub-mediated mechanism responsible for integration of mitochondria into the cellular environment.

## Introduction

Most known functions of ubiquitin (Ub) require its covalent attachment to target proteins (substrates), which is mediated by the sequential action of an E1 activating enzyme, an E2 conjugase and an E3 ligase [1]. E3s have the ability to bind both to an E2 (*via* a RING finger, U box, or HECT “catalytic” domain) and to the substrate –in many E3s, those two binding sites reside in the same polypeptide. In addition, certain RING finger (RNF) proteins can also be part of complexes where substrate recognition is assigned to a separate subunit. In particular, the RNF proteins Rbx1/2 add great diversity to E3s by forming SCF and SCF-like complexes with alternative substrate-recognition subunits, often characterized by the additional presence of an F box, SOCS box, DDB1 or BTB domain [2], [3].

Although the Ub-proteasome system (UPS) is known to be involved in a number of cellular processes, the role of E3s in the regulation and degradation of mitochondrial proteins and in mitochondrial biology is only beginning to emerge. For one thing, until recently, studies of mitochondrial protein degradation had largely focused on the organelle's autonomous and highly conserved proteolytic system, including peptidases and ATP-dependent proteases [4]. In fact, the UPS is not present within mitochondria. However, Ub might conceivably target either proteins that are integral to the mitochondrial outer membrane (MOM) or cytoplasmic regulators of mitochondrial functions. The regulation of mitochondrial dynamics by the UPS was first suggested by work showing that the E3 Rsp5p plays an essential role in this process [5]. Based in particular on studies with *S. cerevisiae* we now know that mitochondrial fusion and fission factors can become modified with Ub or Ub-like proteins [5]–[8]. For example, the steady-state levels of the MOM fusion factor, Fzo1p, are set by ubiquitylation mediated by the E3 Mdm30p followed by proteasomal degradation [6]; the degradation of Fzo1p can also be accelerated by a yet-to-be identified E3 in response to mating pheromone [7]. Modification with the Ub-like protein SUMO promotes the association of the cytosolic fission factor Drp1 with mitochondria and increases its stability, presumably by competition with Ub [8]. In fact, a candidate E3 Ub ligase for Drp1, MarchV/MITOL, which acted as a negative regulator of mitochondrial fission, has been identified recently [9], [10]. MarchV, which until this work was the only transmembrane E3 known to reside in the MOM, binds to Drp1 and mediates its ubiquitylation and destabilization [9], [10]. However, another recent report has proposed the opposite function for MarchV [11], i.e., as a factor *required* for mitochondrial fission. Thus, the picture clearly remains incomplete.

We reasoned that the discovery of new mechanisms of cellular regulation involving the UPS could benefit from the development of genome-wide tools to allow its specific manipulation, such as by interfering with E3 levels. Here we report the annotation of the E3s and substrate-recognition subunits of E3 complexes encoded in the human genome. Based on the annotation information, we generated genome-wide human and mouse cDNA and shDNA E3 collections ready for functional screening. In a cell-based imaging screen for regulators of mitochondrial dynamics using these collections, we identified an E3, FLJ12875, whose role in these processes was made evident both through its ectopic expression and through the knockdown of the endogenous gene. Since this E3 had been previously identified as an NF-κB activator [12] and we found it to localize to mitochondria, we named it MULAN, for Mitochondrial Ubiquitin Ligase Activator of NF-κB. MULAN is anchored to the mitochondrial outer membrane through two transmembrane domains, properly positioned such that its RNF can have access to other UPS components in the cytosol. Both an intact RNF and the E3's mitochondrial localization were required for interfering with mitochondrial dynamics.

## Results

### Genome-wide annotation of the human E3 superfamily

We set out to annotate the minimal complement of genes encoding putative human E3s, based on the presence of signature “catalytic” domains, as well as of domains characteristic of substrate-recognition subunits of multi-subunit RNF-dependent E3s. In addition, we annotated the genes encoding A20 finger proteins and MALT1/paracaspase, which have recently been found to act as E3s [13], [14]. In contrast to two Ub E1 and the estimated fewer than 40 Ub E2 genes (our unpublished observations and [1]) and consistently with the role of E3s in conferring substrate specificity to ubiquitylation, we uncovered ∼617 genes encoding putative Ub E3s after searching a human proteome *superset* and annotating the respective encoding loci. The number of putative E3 genes is thus greater than the number of human genes for protein kinases (518 genes; Tables 1 and S1). We also found that ∼95% of all E3s are RNF-dependent, with RNF domain-encoding genes and multisubunit RNF-dependent E3s represented in similar numbers (309 and 270 genes, respectively). In *S. cerevisiae*, ∼80 genes were found to encode putative E3s –of these, 13 are essential for viability in rich medium, which mirrors the proportion of all yeast genes estimated to be essential under these conditions (17%; Tables 1–3). Thus, a similar fraction of all yeast and human genes are dedicated to encode E3s (1–2%).

**Table 1:** Number of *H. sapiens* and *S. cerevisiae* predicted E3-encoding genes.

Family	human	yeast
RING	300	47
U box	9	2
HECT	28	5
F box	61	21
SOCS box	37	0
BTB	169	3
DDB1-like	3	2
ZnF A20	9	0
other	1	0
***Total***	***617***	***80***
**RING finger E3s**	**human**	**yeast**
single-subunit/U box	309	49
multi-subunit (SCF-like)	270	26
***Total***	***579***	***75***

Top 11 rows: numbers according to individual E3 families. “Other” refers to paracaspase [13]. Bottom four rows: number of single-subunit (including U box) and multi-subunit RNF E3s. ∼95% of both human and budding yeast's E3s are RNF-dependent. Note that the SOCS and A20 E3 families are not represented in budding yeast, and that BTB proteins are relatively underrepresented in yeast compared to humans; in addition, in budding yeast there are nearly twice as many predicted single-subunit as multi-subunit RNF E3s.

**Table 2 pone-0001487-t002:** *S. cerevisiae* RNF E3s and their *H. sapiens* homologs.

*S. cerevisiae* name	*S. cerevisiae* symbol	Human homolog
**APC11**	**YDL008W**	APC11
ASI1	YMR119W	
ASI3	YNL008C	
ASR1	YPR093C	
BRE1	YDL074C	RNF20 (RNF40)
CHF1/DMA1	YHR115C	RNF8
CHF2/DMA2	YNL116W	RNF8
**CWC24**	**YLR323C**	RNF113/ZNF183
FAP1	YNL023C	NFX1 ?
FAR1	YJL157C	
HEX3/SLX5	YDL013W	
HRD1/DER3	YOL013C	AMFR
IRC20	YLR247C	
ITT1	YML068W	RNF14 ?
MAG2	YLR427W	RNF10
**MPE1**	**YKL059C**	RBBP6
NOT4/MOT2/SIG1	YER068W	CNOT4
PEX10/PAS4	YDR265W	PEX10
PEX12/PAS11	YMR026C	PEX12
PEX2	YJL210W	PEX2
PIB1	YDR313C	
PSH1	YOL054W	
RAD16/PSO5	YBR114W	
RAD18	YCR066W	RAD18
RAD5/REV2/SNM2	YLR032W	SMARCA3/HLTF
**RBX1/ROC1/HRT1**	**YOL133W**	RBX1
RIS1/DIS1	YOR191W	
RKR1	YMR247C	ZNF294
RMD5/GID2	YDR255C	
SAN1	YDR143C	
SLX8	YER116C	
SNT2	YGL131C	
**SSL1**	**YLR005W**	GTF2H2
SSM4/DOA10	YIL030C	MARCH family
STE5/HMD3/NUL3	YDR103W	
**TFB3/RIG2**	**YDR460W**	MNAT1
TUL1	YKL034W	
UBR1/PTR1	YGR184C	UBR1
UBR2	YLR024C	UBR2
VPS11/PEP5/END1	YMR231W	VPS11
VPS18/PEP3/VPT18	YLR148W	VPS18
VPS8	YAL002W	FLJ32099
YBR2	YBR062C	
	YDR266C	
	YHL010C	BRAP
	YKR017C	
	YOL138C	

Sources of homology assignments were BLAST searches, Homologene, The Saccharomyces Genome Database, The Sanger Center YOGY database and manual curation. In bold, yeast genes essential for viability. Few human RNF E3s have clear yeast homologs. Indeed, human RNF E3s are often associated with signaling domains that are specific of metazoans (see Table 4).

**Table 3:** Predicted non-RNF E3s of *S. cerevisiae* grouped according to family.

Protein family	Gene name	Gene symbol
**Ubox**	**PRP19**	**YLL036C**
	UFD2	YDL190C
**HECT**	TOM1	YDR457W
	HUL4	YJR036C
	HUL5	YGL141W
	UFD4	YKL010C
	**RSP5**	**YER125W**
**F BOX**	COS111	YBR203W
	SAF1	YBR280C
	MFB1	YDR219C
	**CDC4**	**YFL009W**
	**MET30**	**YIL046W**
		YJL149W
	RCY1	YJL204C
	GRR1	YJR090C
	HRT3	YLR097C
		YLR224W
		YLR352W
	MDM30	YLR368W
	UFO1	YML088W
	SKP2	YNL311C
	DIA2	YOR080W
	ELA1	YNL230C
	AMN1	YBR158W
	**CTF13/CBF3**	**YMR094W**
	RAD7	YJR052W
		YDR306C
		YMR258C
**BTB**		YIL001W
		YDR132C
		YLR108C
**DDB1-like**	**RSE1**	**YML049C**
	**CFT1**	**YDR301W**

Columns 2 and 3: in bold, yeast genes essential for viability.

All but 75 human RNF proteins exhibit at least one other domain that could be identified using publicly available protein domain search databases. The 56 or more types of domains that we found associated with the remaining RNF E3s (Table 4) could play a variety of roles, including mediating interactions with substrate degradation signals (degrons). This variety of domains is consistent both with the diversity of proteins targeted for ubiquitylation and with the diversity of mechanisms involved in their recognition. Domain composition and protein homology have been previously utilized to define subfamilies of RNF proteins. The largest subfamily, TRIM/RBCC, has ∼76 representatives in humans (but none in *S. cerevisiae*) and is characterized by the presence of a B box and a coiled coil ([15] and Table 5). The second largest human RNF subfamily is the RBR/TRIAD [16], with at least 14 members exhibiting two RNFs and an in-between RING (IBR) domain (e.g., Parkin). Interestingly, many RNF E3s are associated with other zinc-binding domains –particularly zinc fingers of the C2H2, C3H1, UBR1 and RBZ types (Table 4). Alignment of the RNF domain alone did not reveal any new subfamilies of RNF proteins (unpublished observations).

**Table 4:** Protein domains predicted in RNF E3s.

Domain	Number of RNF Genes	Representatives
Transmembrane	46	AMFR, BFAR, BFP, MULAN, DCST1, IBRDC3, PTD016, GOLIATH, RNF103, RNF121, RNF122, RNF128, RNF13, RNF133, RNF139, RNF145, RNF148, RNF149, RNF150, RNF152, RNF167, RNF170, RNF175, RNF180, RNF182, RNF183, RNF185, RNF186, RNF19, RNF26, RNF5, ZFPL1, LL441061, ZNRF4, TRIM59, SYVN1, MIR, MARCH1, MARCH2, MARCH3, MARCH4, MARCH5, MARCH6, MARCH8, MARCH9, RFP2
ZnF_C2H2	10	DPF1, HAKAI, ZNF598, FLJ2573J, UB1-D4, RNF166, ZnF313, RNF125, RAG1, ZNF645
Protease-Associated (PA)	9	RNF13, RNF130, RNF133, RNF148, RNF149, RNF150, RNF128, RNF167, ZNRF4
ZnF_TRAF	8	PDZRN3, RNF151, TRAF2, TRAF3, TRAF4, TRAF5, TRAF6, TRAF7
MATH	6	TRAF2, TRAF3, TRAF4, TRAF5, TRAF6, TRIM37
ZnF_C3H1	6	MKRN1, MKRN2, MKRN3, MNAB, RNF113A, RNF113B
BIR	5	cIAP1, cIAP2, BIRC4, livin, BIRC8
TPR	5	RAPSYN, TPRD1, LL286495, RNF127, RNF105
Ankyrin repeats	4	ANKIB1, MIB1, MIB2, BARD1
KH	4	LL92312, RKHD1, RKHD2, RKHD3
PDZ	4	LNX, LNX2, PDZRN3, PDZRN4
Swi-related	4	SHPRH, SMARCA3, MDMX, MDM2
WWE	4	DTX1, DTX2, DTX4, RNF146
LON	3	LONRF1, FLJ45273, RNF127
PEX	3	PEX10, PEX12, PEX2
SH2 variant	3	c-CBL, CBL-B, CBL-3
Ubiquitin-like	3	PARKIN, UHRF1, UHRF2
WD40	3	COP1, RFWD3, TRAF7
ZnF_C2HC	3	RBBP6, ZNRF1, ZNRF2
ZnF_RBZ	3	MDMX, RNF31, RBCK1
DEXDc	3	ATRX, SHPRH, SMARCA3
NEUZ	3	LINCR, NEURL, LL391849
ZnF_ZZ	3	MIB1, MIB2, ZSWIM2
BRCT	2	BARD1, BRCA1
FHA	2	CHFR, RNF8
SH3	2	SH3MD2, SH3RF2
ZnF_UBR1	2	UBR1, UBR2
ZnF_NFX	2	HOZFP, NFX1
SAM	2	BFAR, LRSAM1
CARD	2	cIAP, cIAP2
RWD	2	RNF14, RNF25
RPT	2	RNF187, LL390358
HELICc	2	SHPRH, SMARCA3
SRA	2	UHRF1, UHRF2
LRR	1	LRSAM1
RRM	1	cNOT4
VWA	1	SSL1
ZnF_UBP	1	BRAP
CUE	1	AMFR
RPT	1	ATRX
AAA	1	LL57674
MAT1	1	MAT1
B41	1	MIR
Kinase	1	MEKK1
SAP	1	RAD18
ZnF_CHY	1	RCHY1
IQ	1	RNF32

See Pfam/SMART for domain definitions. This list is not exhaustive and is subject to rapid change as novel domain-defining algorithms are included in the databases. Shown are the number of RNF-encoding genes that also encode the indicated domains. Evidently, a given domain can be present more than once in certain proteins. In addition to those listed, we also found the following domains, almost exclusively in the TRIM subfamily (protein numbers in parentheses): B box (72), SPRY (58), PRY (28), BBC (11), FN3 (7), BROMO (4), PHD (3), IG FLMN (3), NHL (3), ARF (1).

**Table 5:** Major RNF E3 subfamilies.

Protein Family	Number of Genes	Representatives
TRIM/RBCC	76	TRIML1,TRIM10, TRIM11, TRIM15, TRIM17, TRIM2, TRIM21, TRIM22, TRIM23, TRIM25, TRIM26, TRIM28, TRIM3, TRIM31, TRIM32, TRIM33, TRIM34, TRIM35, TRIM36, TRIM37, TRIM38, TRIM39, TRIM4, TRIM40, TRIM41, TRIM42, TRIM43, TRIM45, TRIM46, TRIM47, TRIM48, TRIM49, TRIM49L1, TRIM49L2, TRIM49L3, TRIM5, TRIM50, TRIM51, TRIM39L, TRIM52, TRIM54, TRIM55, TRIM56, TRIM58, TRIM59, TRIM6, TRIM60, TRIM61, TRIM62, TRIM63, TRIM64, TRIM65, TRIM67, TRIM68, TRIM69, TRIM7, TRIM72, TRIM73, TRIM74, TRIM75, TRIM8, TRIM9, RFPL4B, LL390231, MID1, MID2, PML, RFP, RFP2, RFPL1, RFPL3, RNF135, RNF39, TIF1, RFPL4A, LL399937
TRIAD/RBR	14	TRIAD3, C20orf18, IBRDC1, ANK1B1, ARI, ARI2, IBRDC2, IBRDC3, PARKIN, PARC, RNF14, RNF144, RNF19, RNF31
MARCH	9	MARCH1, MARCH2, MARCH3, MARCH4, MARCH5, MARCH6, MARCH8, MARCH9, LOC441061
GOLIATH	9	GOLIATH, RNF13, GRAIL, RNF133, RNF148, RNF149, RNF150, RNF167, ZNRF4
POLYCOMB	8	PCGF1, PCGF2, PCGF3, PCGF5, PCGF6, PCGF4, RING1, RNF2
TRAF	7	RNF151, TRAF2, TRAF3, TRAF4, TRAF5, TRAF6, TRAF7
DELTEX	5	DTX1, DTX2, DTX4, DTX3L, RNF146, DTX3
IAP	5	cIAP1, cIAP2, BIRC4, livin, BIRC8
UBR	4	UBR1, UBR2, ZNF650, LOC51136
PRAJA	4	PJA1, PJA2, RNF126, ZNF364
RKHD	4	RKHD1, RKHD2, RKHD3, LOC92312
NEURALIZED	3	LL93082, NEURL, LL391849
PEX	3	PEX2, PEX10, PEX12
MAKORIN	3	MKRN1, MKRN2, MKRN3
LON-RF	3	FLJ45273, RNF127, LONRF1
SIAH	3	SIAH1, SIAH1L, SIAH2
CBL	3	c-CBL, CBL-B, CBL-3, (HAKAI?)

Proteins lacking a RNF domain are not listed (e.g., TRIM14, TRIM16, TRIM29). Complete or partial annotation of some of the listed families had been previously reported: general [3], [32], TRIM [15], [33], [34], RBR [16], [35], [36], BTB [37], [38] and F box [39].

### Functional genomics screening and identification of MULAN, an E3 that regulates mitochondrial dynamics

The discovery of new mechanisms of cellular regulation involving the Ub-proteasome system (UPS) could benefit from the development of functional genomics tools that allow its specific manipulation, such as by interfering with E3 levels. With this in mind, we hit-picked cDNA and plasmid-encoded RNAi (shDNA) expression constructs representing nearly the entire human and mouse E3 families from publicly available genome-wide collections, and individually arrayed these constructs in 384-well plates for functional screens [17].

**Figure 1:**
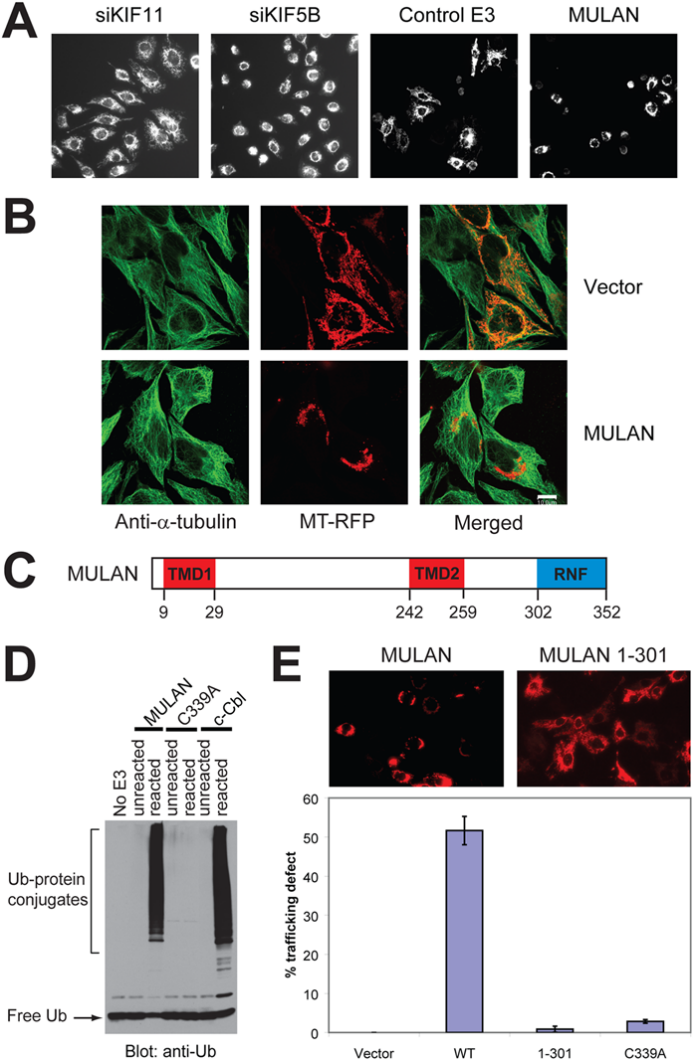
Identification of MULAN as a novel regulator of mitochondrial dynamics. A) *Left 2 panels:* Mitochondrial perinuclear clustering (a consequence of defective trafficking) results from siRNA-mediated knockdown of KIF5B, but not of KIF11. HeLa cells were transfected with the indicated siRNAs and mitochondria were visualized with MitoTracker Red. *Right 2 panels:* A cell-based imaging screen of E3 cDNA and shDNA genome-wide collections led to the identification of MULAN, a RING finger protein whose ectopic expression resulted in mitochondrial perinuclear clustering and fragmentation. For the screen, HeLa cells were co-transfected with mitochondrial-targeted RFP (MT-RFP) and with the E3 collections. “Control E3” refers to a random E3 cDNA which did not affect mitochondrial dynamics in the screen. B) Mitochondrial perinuclear clustering in response to MULAN ectopic expression apparently does not result from disruption of the microtubule network. HeLa cells were co-transfected with MT-RFP and either vector control or MULAN wild type cDNA. Cells were immuno-stained with anti-α-tubulin antibody to visualize the microtubule network. Green, tubulin staining; red, mitochondria. C) MULAN's schematic domain structure. Amino acid numbers indicated below. TMD, transmembrane domain. RNF, RING finger. D) MULAN's RNF has *in vitro* E3 activity. Reactions utilized GST fusions with the MULAN or c-Cbl RNFs as E3s. Activity was dependent on an intact RNF, since it was not detected in reactions using the MULAN C339A RNF mutant. c-Cbl's RNF was used as a positive control. Negative controls were reactions lacking an E3 (No E3) or reactions added to SDS sample buffer at t = 0 (unreacted). E) MULAN's RNF is required for the regulation of mitochondrial dynamics. NIH3T3 cells were transfected with vector, wild type MULAN cDNA or RNF-mutant cDNAs, together with MT-RFP. Cells were fixed at 24 h post-transfection and the fraction of RFP-positive cells exhibiting perinuclear-clustered mitochondria was scored (% trafficking defect). Data represent the average of at least 350 cells per condition, from 3–5 random 20× fields. Results are representative of several experiments.

In one such screen, we set out to identify novel E3s that regulate mitochondrial dynamics. Mitochondria divide, fuse and move around cells [18]–[20], processes whose relevance is underscored by the observation that mutations in the cognate proteins can lead to disease, in particular neuronal dysfunction [19]. Mitochondria utilize the cytoskeleton as tracks for directional movement –in mammalian cells, they are distributed mainly along microtubules, with kinesins (KIF) 1B and 5B having been identified as the anterograde (plus end) trafficking motors [21]. A phenotype that is characteristic of defective mitochondrial anterograde trafficking is the perinuclear clustering of the organelle, which we observed following transfection of HeLa cells with siRNA against KIF5B (a gift of Dr. Jiang Wei, Burnham Institute; [22]); in contrast, mitochondrial trafficking was not impaired in cells transfected with siRNA to KIF11, which has not been implicated in mitochondrial trafficking (Fig. 1A).

Having validated a morphology-based imaging assay for mitochondrial dynamics, we next screened our E3 collections by transfecting HeLa cells together with a plasmid encoding a mitochondrial-targeted red-fluorescent protein (MT-RFP, consisting of RFP fused to the presequence of human cytochrome c oxidase subunit VIII). This allowed visualization of mitochondria specifically in E3-transfected cells. Following hit-picking and reconfirmation of the results from the primary screens, we uncovered MULAN, a putative E3 whose ectopic expression led to defects in both mitochondrial morphology and subcellular distribution (Fig. 1A). The phenotype of HeLa cells ectopically expressing MULAN is characterized by the fragmentation of otherwise long, tubular-shaped mitochondria (Figs. 1A and 1B). In addition, as in the KIF5B knockdown, mitochondria in MULAN-transfected cells clustered around the perinuclear region. These phenotypes were MULAN dose-dependent (Fig. S1) and were observed with all cell lines examined: HeLa, 293, NIH3T3, COS7, H9c2 and HL1 (unpublished observations). Notably, the phenotypes resulting from MULAN ectopic expression were manifested rapidly, within 24 h of transfection.

The effects of MULAN on mitochondrial dynamics could be indirect, e.g., as a result of the disruption of the microtubule network. To examine this possibility, HeLa cells were co-transfected with MT-RFP and either MULAN or the vector control, and microtubules were visualized by staining with α-tubulin antibody. The results showed no obvious differences in the microtubule network between vector- and MULAN cDNA-transfected cells (Fig. 1B). Thus, the altered mitochondrial distribution in cells ectopically expressing MULAN was apparently not due to microtubule disruption. However, these results do not rule out the possibility that the E3 exerts more subtle effects on microtubules that could not be detected with these analyses.

MULAN is a 40-kDa protein with orthologs identified from flies to vertebrates, and in plants. As MarchV, it does not have an obvious ortholog in yeast. Reciprocally, Mfb1p and Mdm30p, budding yeast F-box E3 subunits that are essential for mitochondrial dynamics, are apparently not conserved in higher eukaryotes [6]. One possibility to explain the lack of conservation of such critical regulators of what is thought to be an essential process for both yeast and human cells is that there are important distinctions between the molecular mechanisms underlying mitochondrial dynamics in these organisms. Of relevance is the observation that mitochondrial trafficking in yeast largely relies on the actin cytoskeleton network, while in mammalian cells mitochondria are transported predominantly along microtubules [18], [21]. Moreover, E3 regulators of mitochondrial dynamics could function as mediators of the response to organism-specific signaling, as is the case of organellar trafficking along long motor neuron axons, a process regulated both by synaptic activity and by secreted molecules such as NGF and TNF (e.g., [21]).

MULAN exhibits two predicted transmembrane domains (TMDs) as well as an evolutionarily conserved C-terminal RNF domain (Fig. 1C and Fig. S1A, B). To determine whether MULAN is a *bona fide* E3, a GST fusion with its RNF was tested for the ability to mediate ubiquitylation in reactions utilizing the promiscuous E2, Ubc4. The RNF from MULAN, under these conditions, was nearly as potent for ubiquitylation as that of c-Cbl (Fig. 1D). As is often the case in this type of assay, GST blots revealed that reaction products consisted of, at least in part, the auto-ubiquitylated GST-RING finger protein itself, and their formation required E1, E2, E3 and Ub (unpublished observations). We also examined whether the integrity of the MULAN RNF domain was required for E3 activity. C339, one of the Cys residues that are essential for maintaining the RNF structure through zinc coordination, was mutated to Ala, resulting in abolished E3 activity (Fig. 1D). This result led us to ask whether the mitochondrial morphology phenotypes associated with expression of MULAN were also dependent on an intact RNF. NIH3T3 cells transfected with MULAN wild-type or with RNF mutant constructs were analyzed for mitochondrial distribution (Figs. 1E and S1D). While mitochondria in *ca*. 52% of wild type MULAN-transfected cells presented a trafficking defect, this number was markedly reduced in cells expressing either the RNF-deleted E3 (1-301; 1%), or the C339A mutant (3%) (Fig. 1E). These mutants were expressed at similar or higher levels than the wild-type (Figs. S1D and S2A) and their lack of effect on mitochondrial dynamics was even more evident when normalized to the wild-type expression level (Fig. S1D). Thus, the simplest interpretation of these results is that regulation of mitochondrial dynamics by MULAN is dependent on its E3 activity. The results also suggest that the mitochondrial phenotype that we observe due to MULAN expression is not just a non-specific effect due to increased expression of *any* protein on the mitochondrial surface.

### *Endogenous* MULAN regulates mitochondrial dynamics

**Figure 2:**
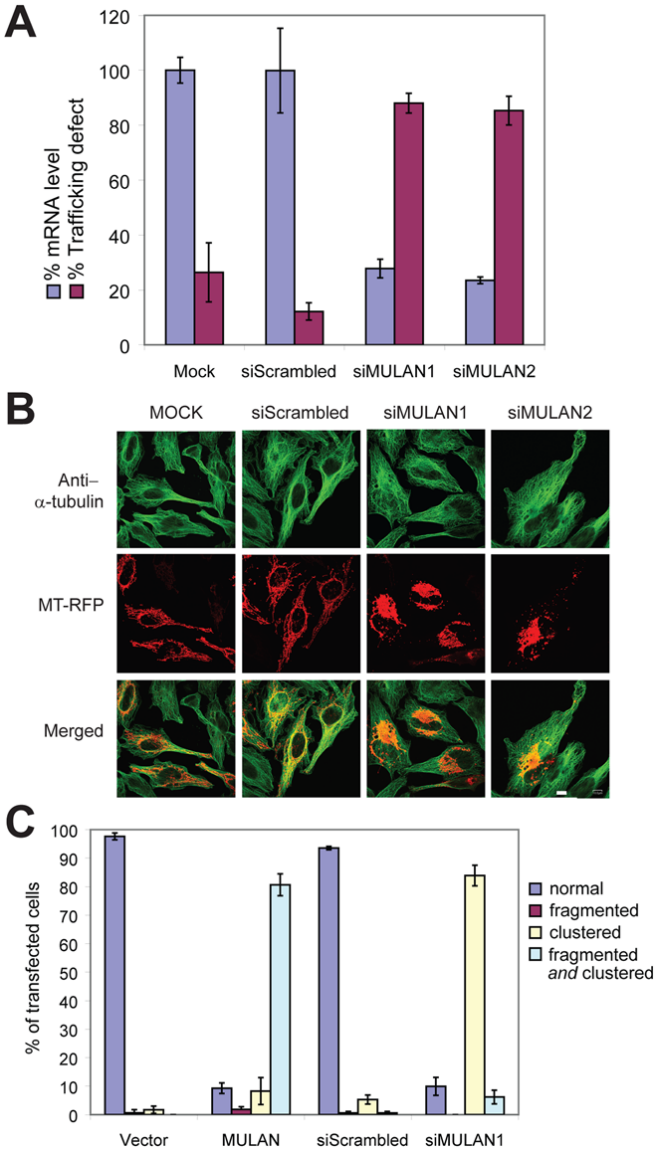
Knockdown of endogenous MULAN perturbs mitochondrial dynamics. A) *Blue bars*: MULAN mRNA knockdown. RNA was extracted from HeLa cells 48 h after transfection with siRNA oligos targeting two different MULAN sequences, or with a negative control siRNA (siScrambled). Knockdown efficiency was determined by quantitative real-time RT-PCR. Results were normalized to the level of the 36B4 mRNA, which was amplified in the same multiplex reaction, as an internal control. *Purple bars*: MULAN knockdown leads to mitochondrial perinuclear clustering. HeLa cells were transfected with the indicated siRNAs on day 1, followed by MT-RFP on day 2. Cells were fixed on day 3 and the percentage of RFP-positive cells with perinuclear-clustered mitochondria was scored. B) The microtubule network is apparently intact in MULAN-knockdown cells. As in “A”, except that cells were fixed and stained with anti-α-tubulin antibody for analysis (green). C) Both MULAN ectopic expression and endogenous knockdown indicate its role in mitochondrial dynamics. For ectopic expression, HeLa cells were transfected with vector or MULAN wild type cDNA together with MT-RFP. Cells were fixed for analysis 24 h post-transfection. For siRNA-mediated knockdown, cells were transfected with siScrambled or siMULAN1 on day 1 and with MT-RFP on day 2. Cells were fixed on day 3. Mitochondrial fragmentation and clustering were quantitated under 100× field. At least 100 cells were counted per condition and data represent average of three independent transfections.

To confirm that *endogenous* MULAN plays a role in mitochondrial dynamics, we generated two different siRNA, siMULAN1 and siMULAN2, which led to greater than 70% reduction of the endogenous mRNA level two days after transfection of HeLa cells (Fig. 2A; knockdown was also observed at the protein level, Fig. S2C). The phenotype of MULAN knocked-down cells exhibited similarities to that of cells ectopically expressing the E3, which is not unprecedented (e.g., [23], [24]) and could be due to a dominant negative effect of the ectopically expressed protein (Fig. 2 and Fig. S3). Mitochondria in 80–90% of MULAN siRNA-transfected cells were clustered around the nucleus, although clustering was less compact compared to that seen in response to ectopic MULAN expression; in contrast, only 5–12% of cells transfected with the control non-silencing siRNA exhibited this phenotype (siScrambled; Figs. 2A and 2C). Immunostaining with anti-α-tubulin antibody in cells knocked down for MULAN did not reveal an obvious disruption of the microtubule network (Fig. 2B). The fact that the knockdown phenotype was specific to siRNA against MULAN and that it was observed with two different siRNA sequences argues against an off-target effect and confirms a role for MULAN in the regulation of mitochondrial dynamics.

With regard to mitochondrial morphology, the phenotype of MULAN-knockdown cells was not as evident as for cells ectopically expressing the E3 (Fig. 2C and Fig. S3). In the latter, mitochondrial clustering is accompanied by a high degree of fragmentation and by the collapse of the mitochondrial network, leaving little discernible tubular structure remaining; in MULAN-knockdown cells, tubular-shaped mitochondria remain visible and the increase in fragmentation relative to control cells is not as marked. These results suggest that the increased mitochondrial fragmentation in response to MULAN ectopic expression could represent either a gain-of-function phenotype or a secondary consequence of severely disrupted mitochondrial trafficking.

### MULAN is a MOM protein with a C-terminal cytosolic-facing RNF

**Figure 3:**
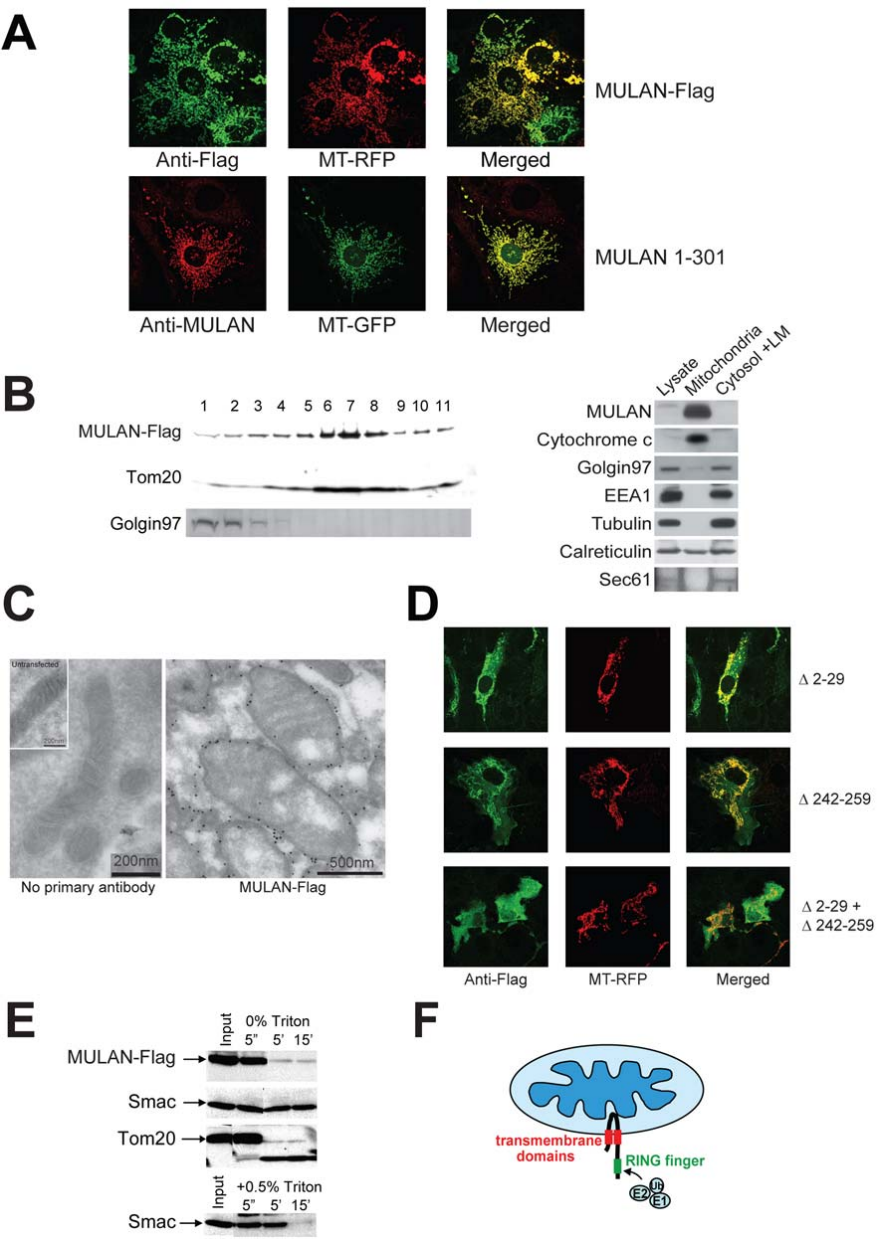
MULAN is a mitochondrial outer membrane (MOM) protein with a cytosolic-facing C-terminal RING-finger. A) MULAN colocalizes with MT-RFP/MT-GFP. NIH3T3 cells were transfected with Flag-tagged MULAN or untagged MULAN 1-301, together with MT-RFP (top panels) or MT-GFP (bottom panels), followed by immunostaining with antibody against Flag (top; green) or MULAN (bottom; red). B) *Left panel:* MULAN-Flag co-sediments with the mitochondrial protein, Tom20, in sucrose gradient. 293 cells transfected with MULAN-Flag were subfractionated by sucrose gradient. MULAN-Flag, the MOM protein Tom20, and Golgin 97 in each fraction were detected by western blot. *Right panel:* endogenous MULAN co-fractionates with mitochondria in sucrose gradient. 293F cells were Dounce-homogenized and centrifuged at 750×g to pellet nuclei and unbroken cells. The post-nuclear supernatant was centrifuged at 12,500×g to obtain the heavy membrane (HM) and the cytosolic/light membrane (LM) fractions. The HM fraction was subjected to sucrose gradient to further purify mitochondria. Equal protein amounts (40 µg) were fractionated by SDS-PAGE and blotted with anti-MULAN antibody to detect endogenous MULAN. Cytochrome c, Golgin 97, EEA1 and tubulin were used as markers for mitochondria, Golgi, endosomes and cytosol, respectively. Calreticulin and Sec61 were both used as ER markers. C) Immuno-electron microscopy of MULAN. COS7 cells transfected or not with MULAN-Flag were fixed and stained with anti-Flag antibody followed by gold-conjugated secondary antibody for immuno-EM analysis. Left panel: BSA only (no primary antibody) control; inset: mitochondrion from an untransfected cell. Right panel: mitochondria expressing MULAN-Flag. Scale bars are indicated. D) MULAN's predicted transmembrane domains (TMDs) mediate localization to mitochondria. NIH3T3 cells were co-transfected with Flag-tagged MULAN deletion mutants (green) and MT-RFP (red). Deletion of TMD1 (MULAN Δ2-29; top row) or of TMD2 (MULAN Δ242-259; middle row) led to partial mislocalization of MULAN. The combined deletion of both TMDs led to complete mislocalization of MULAN to the cytosol (MULAN Δ2-29+Δ242-259; bottom row). E) MULAN is a mitochondrial outer membrane (MOM) protein. Mitochondria from 293 cells transfected with MULAN-Flag were purified by sucrose gradient. Intact mitochondria were treated with trypsin for the indicated time, in the presence or absence of Triton X-100. The MULAN C-terminus was readily susceptible to trypsin digestion in intact mitochondria, indicating that it sits in the MOM facing the cytosol. Controls are the MOM protein, Tom20, and the intermembrane space protein, Smac. Smac only becomes sensitive to trypsin upon lysis of mitochondria with 0.5% Triton X-100 (lower panel). F) Topological model for MULAN on the MOM, indicating its transmembrane domains (red) and RNF (green). The cytosolic-exposed RNF can have access to the remaining components of the Ub system.

To begin elucidating the mechanism by which MULAN affects mitochondrial dynamics, we determined its subcellular localization. Earlier results had already revealed that the protein's distribution was reminiscent of mitochondrial staining patterns. Several lines of evidence indeed indicate that MULAN is a mitochondrial protein: (*i*) the signal of C-terminal Flag-tagged MULAN *completely* overlapped with that of co-transfected MT-RFP (Fig. 3A, upper panels). The *untagged* MULAN 1-301 protein, whose levels were high enough to be detected by immunocytochemistry using anti-MULAN antibody, also colocalized with MT-GFP (Fig. 3A, lower panels). Thus, both an untagged and the C-terminal tagged E3 co-localized with mitochondria. The specificity of MULAN localization is also revealed by the observation that neither protein colocalized with the Golgi marker lectin-Alexa 488 (Fig. S1E); (*ii*) lysates of 293 cells overexpressing MULAN-Flag were subjected to centrifugation in sucrose gradient. Gradient fractions were run in SDS-PAGE and analyzed by western-blot with different antibodies, revealing that MULAN's sedimentation was similar to that of the mitochondrial protein Tom20 but not of the Golgi marker, Golgin 97 (Fig. 3B, left panel); (*iii*) detergent-free lysates of untransfected 293F cells were first fractionated by differential centrifugation. Mitochondria in the heavy membrane (HM) fraction were further purified by sucrose gradient centrifugation. Samples of the whole cell extract, “mitochondrial” fraction, and cytosolic and light membrane (LM) fraction were normalized for protein amount and analyzed by western-blot using antibodies against cytochrome c, Golgin 97, calreticulin/Sec61, tubulin or EEA1 (markers for mitochondria, Golgi, ER, cytosol and endosomes, respectively; Fig. 3B, right panel). Consistently with the previous observations, the results show that *endogenous* MULAN was abundant in the fraction enriched for mitochondria, but was not detected in the fraction enriched for Golgi, ER, cytosol and endosomes. Although some Golgi material contaminated the “mitochondrial” fraction, immunocytochemistry analyses also revealed that MULAN-Flag did not detectably colocalize with lectin in intact cells (Fig. S1E). In the case of the ER, there was also contamination of the mitochondrial fraction with soluble ER luminal material (calreticulin) and the ER signal in immunocytochemistry was too disperse to allow complete resolution from mitochondria (Fig. S1F). However, we do not expect a significant fraction of MULAN to be localized to the ER, since this organelle's membrane-resident marker Sec61 was not detected in the purified mitochondrial fraction enriched for MULAN, and MULAN was not detected in the LM fraction enriched for the ER (Fig. 1B); (*vi*) lastly, immuno-electron microscopy analyses demonstrated that the MULAN signal concentrated specifically around the circumference of mitochondria (Figs. 3C and S1). The finding that MULAN localizes to mitochondria is interesting because only one other transmembrane E3 has been shown to reside in this organelle [9], [11]. These results suggest that MULAN's effects on mitochondrial dynamics are direct.

We then set out to determine the localization and topology of MULAN within mitochondria. To confirm that MULAN's predicted TM domains are functional, we generated MULAN-Flag mutants with deletions of sequences encompassing the TMD1 (amino acids 9-29), TMD2 (amino acids 242-259), or both. Fig. 3D shows that deletion of either TMD partially affected the co-localization of MULAN with MT-RFP, and that deletion of both TMDs completely mistargeted the protein. These results are consistent with the prediction that MULAN is a transmembrane protein, but do not distinguish whether it sits at the outer or the inner mitochondrial membranes. Access to other components of the Ub system would require MULAN to be localized to the MOM with the RNF domain facing the cytosolic side. To test this possibility, we performed a limited protease susceptibility experiment with purified mitochondria. 293 cells expressing MULAN-Flag were homogenized in isotonic sucrose buffer and intact mitochondria were isolated by sucrose gradient purification. Mitochondria were incubated with or without trypsin for various times and used for western blots to detect MULAN-Flag, the intermembrane space protein Smac, and the MOM protein Tom20. The results (Fig. 3E) revealed that MULAN's C-terminal Flag tag was readily susceptible to trypsin digestion, its signal disappearing within 5 min of treatment. Similar results were obtained with Tom20, while Smac remained trypsin-resistant unless mitochondria were permeabilized with 0.5% Triton X-100 prior to addition of the protease (Fig. 3E, lower panel). Together, the results presented so far suggest that MULAN is MOM-anchored through two transmembrane domains, exposing a short N-terminus and the RNF-containing C-terminus to the cytosol. This model predicts that its RNF should have access to the remaining components of the Ub system and thus function as a *bona fide* E3 *in vivo* (Fig. 3F).

### Modulation of mitochondrial dynamics by MULAN requires its proper localization to the organelle

**Figure 4:**
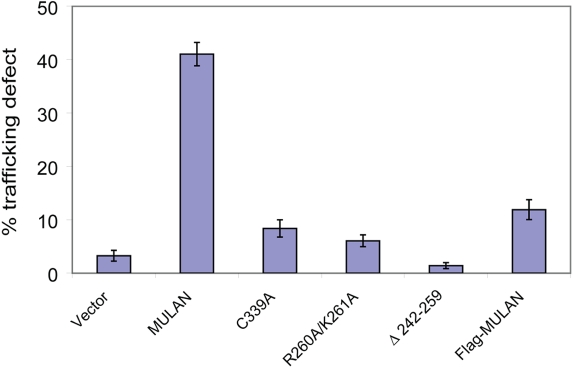
Modulation of mitochondrial dynamics by MULAN requires its proper localization to the MOM. NIH3T3 cells were transfected with wild type or mitochondrial localization-defective MULAN mutants, together with MT-RFP. C339A is the RING finger-mutant control. Cells were fixed 24 h post-transfection and the percentage of RFP-positive cells with perinuclear-clustered mitochondria was determined.

We next investigated the mechanisms that target MULAN to mitochondria and found that, as for certain other MOM proteins, transmembrane sequences with moderate hydrophobicity and net positive charge in flanking regions, known as signal-anchor or TMD+ domains are involved ([25] and Figs. S4 and S5, Text S1). For example, mutation of two positively charged residues C-terminal to TMD2 (R260A/K261A) led to MULAN's targeting to the endoplasmic reticulum (Fig. S4C); MULAN's N-terminus also seemed to influence proper targeting, since an N-terminal Flag tag led to the protein's cytosolic localization (Fig. S4D). To determine whether modulation of mitochondrial dynamics by MULAN requires its proper localization to the organelle, the TMD2-deleted mutant, the R260A/K261A mutant, as well as N-terminal Flag tagged protein were evaluated for the ability to promote perinuclear clustering of mitochondria. The results in Fig. 4 show that mistargeted MULAN mutants were defective in eliciting perinuclear clustering, despite that all were expressed at levels comparable or higher to the wild-type (Fig. S2B). Likewise, MULAN's isolated cytoplasmic domain (amino acids 260-352) had no effect on mitochondrial distribution or morphology (unpublished observations). Interestingly, although the TMD2-deleted mutant (Δ242-259) still remained partially localized to mitochondria, it was completely defective in promoting perinuclear clustering of the organelle, perhaps suggesting that an intact MULAN structure is required for interaction with other components of its pathway. The results above are consistent with a model in which MULAN's regulation of mitochondrial dynamics involves signaling effectors that are either integral to, or can peripherally associate with, mitochondria.

### MULAN is most highly expressed in the human heart

**Figure 5:**
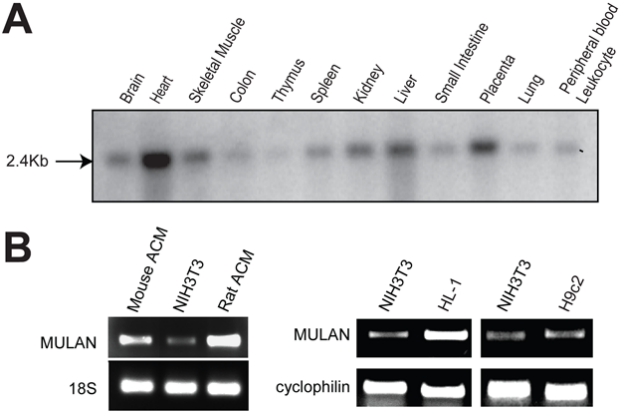
MULAN is most highly expressed in the human heart. A) Full-length human MULAN cDNA was used as a probe for hybridization to northern-blot of RNA from multiple human tissues. A single 2.4Kb band was detected for MULAN mRNA, in various tissues. B) RT-PCR of MULAN mRNA showed higher expression in mouse and rat adult primary cardiomyocytes (ACM) and in the mouse HL-1 cardiomyocyte cell line, compared to mouse NIH-3T3 cells and to the rat skeletal muscle-like cell line H9c2. 18S rRNA and Cyclophilin B were used as controls.

To determine MULAN's expression pattern, we used MULAN cDNA to probe a Northern blot of RNA from several human tissues. The result revealed a single, 2.4-Kb mRNA in most human tissues tested, with highest levels in the heart (Fig. 5A). Consistently, semi-quantitative RT-PCR analyses revealed that MULAN is also expressed at high levels in isolated mouse and rat primary cardiomyoctyes (Fig. 5B, left panels) as well as in the adult mouse cardiomyocyte cell line HL-1 when compared to NIH3T3 cells or to the rat skeletal muscle-like cell line H9c2 (Fig. 5B, right panels).

## Discussion

In this study we performed the genomic annotation of the human E3 superfamily and identified *ca*. 617 genes encoding putative E3s and substrate recognition subunits of multimeric E3 complexes. While there is no definitive proof that all those genes encode *bona fide* E3s or E3 subunits, we note that most of the annotated domains have a general or *quasi* general function in mediating ubiquitylation. This is certainly true for RNF-dependent E3s, which account for ∼95% of all human E3s. To our knowledge, even in the case of RNF proteins whose E3 activity has not been directly demonstrated, there is evidence for an involvement in ubiquitylation. For example, the RNF protein Bard1 seems to be inactive for ubiquitylation alone but stimulates the E3 activity of its heterodimeric partner, BRCA1 [26].

Only a small fraction of all E3s has been functionally characterized. Despite that, E3s have already been directly implicated in many biological processes and diseases. Well known examples are deregulation of the E3s BRCA1, Mdm2, VHL and Skp2 in various cancers, and Parkin in Parkinson's disease. As a follow-up of a genomic annotation of the E3 superfamily, we have generated plate-arrayed, genome-wide cDNA and shDNA E3 collections that can serve as tools for the functional analysis of E3 function. Because of the reduced size of such “protein family” collections relative to complete genomic collections of tens of thousands of entities, the quality of screens can be improved by testing samples in replicates, for example. It also makes it more feasible to repeat screens and change variables, such as by using different cell lines (e.g., to knock-down regulators whose mRNA expression is cell type-specific). We expect that this tool will lead to the discovery of novel mechanisms of biological regulation by Ub as well as to new candidate drug targets. The cell-based imaging screen that we described in this work exemplifies how our collections could be utilized to discover novel E3s and to assign novel functions to known E3s. Other screen readouts are conceivable –for example, it should be possible to uncover E3s that target a substrate of interest by using a substrate-reporter fusion protein. Furthermore, screening could be applied to a transcriptional reporter-based assay for a signaling pathway.

Mitochondrial dynamics is an essential aspect of the organelle's function. We reported above the identification of a novel regulator of this process, the MULAN E3 ligase. Three general lines of evidence strongly suggest that the role we uncovered for MULAN in the regulation of mitochondrial dynamics is specific –the protein's localization, as well as its ectopic expression and loss-of-function phenotypes. As a consequence of MULAN's knockdown, the marked phenotype was a profound effect on the distribution of mitochondria. This phenotype is thus distinct from the one resulting from knockdown of MarchV/MITOL, the only other mitochondrial E3 described so far [9], [10]. In the latter case, increased mitochondrial fission and unaltered mitochondrial distribution were observed, suggesting that MULAN and MarchV play different roles in mitochondria (but see [11]).

How does MULAN work? Its effects on mitochondrial dynamics are likely to be direct, since MULAN localizes to mitochondria and those effects required MULAN's proper localization to the organelle. We presume that it is less likely that MULAN's primary role is on fusion or fission, since these processes were unaffected upon MULAN depletion. Moreover, co-expression of either an inhibitor of mitochondrial fission (the GTPase-defective, dominant negative Drp1^K38A^ mutant [27]) or the mitochondrial fusion factors Mfn1, Mfn2 or OPA1 failed to reduce the mitochondrial fragmentation induced by MULAN ectopic expression suggesting that MULAN's effects on mitochondrial morphology are unrelated to the canonical fusion or fission pathways (unpublished observations). Instead, the results presented are consistent with a role for MULAN at the level of trafficking regulation –both its ectopic expression and knockdown led to altered mitochondrial distribution, without having apparent effects on the microtubule network. MULAN is an active E3, and its effects on mitochondrial dynamics also required an intact RNF domain. Clearly, the identification of MULAN's substrates, effectors or interacting proteins should help determine its primary function.

Besides being the primary organelle for energy production, mitochondria also play important roles in intracellular signaling –most studied have been their roles in programmed cell death. Signaling responses can also be triggered by mitochondrial systems monitoring protein quality (e.g., [28]). In higher eukaryotes, mitochondria signal stress to the nucleus through still poorly characterized pathways involving the transcription factors CHOP, C/EBPβ or NF-κB [28], [29]. So far, the most definitive link connecting NF-κB activation to mitochondria is through MAVS, a MOM-resident protein that participates in the host innate immune response to viral infection [29]. However, the mechanisms of NF-κB activation by MAVS remain unclear. MULAN had been previously identified as an NF-κB activator [12] and we were able to confirm those findings in our laboratory (unpublished observations). The discovery that MULAN is an E3 anchored to the MOM thus provides an important new link between mitochondria and NF-κB activation and an exciting new opportunity for elucidating how these organelles become integrated into the cellular environment.

## Materials and Methods

### Genomic annotation

To identify putative E3-encoding genes, the human proteome deduced from a *superset* combining 5 databases [RefSeq peptides, predicted transcripts of the human genome assembly #36, Ensembl release 39, transcripts and corresponding proteins predicted by Celera (release R27), and a 2006 version of UniProt] was searched with Hidden Markov Models designed for the individual domains, using HMMER 2.3.2 (based on the Interpro v.13.1, including but not limited to Pfam database v.20). The related PHD, PIAS and LIM domains were excluded from the analyses. Iterative BLAST searches (v.2.2.12) of the UniGene, RefSeq and Ensembl were used to compile a complete list of “synonyms” (alternative accession numbers), and using database cross-references subsequently unified by non-redundant identifiers –EntrezGene IDs (or a Celera hCG in the absence of an Entrez gene). In some cases, domain assignment or verification involved manual comparison to the predicted domains of the proteins' orthologs listed in Homologene. In a limited set, some manual editing was also used to analyze and combine *de novo* gene model predictions from genomic sequences (using the Genscan program, Celera and Ensembl gene models) with split predictions into a full-length gene model. Prediction of additional domains in RNF E3s was performed with SMART (http://smart.embl-heidelberg.de/).

### Plamids and constructs

Human full length MULAN cDNA (GenBank accession no. NM_024544) constructs in the pME vector were previously described [12]. Mouse full length MULAN cDNA (GenBank accession no. NM_026689) was obtained from the Mammalian Genome Collection. Vectors used to generate tagged MULAN constructs were p3xFlag-CMV-14 (Sigma, Saint Louis, MO), pFlag, EGFP-N3 and EGFP-C2 (Clontech, Mountain View, CA). Point mutations were created using the QuickChange site-directed mutagenesis kit (Stratagene, La Jolla, CA). Internal deletions were generated using the inverse PCR method. All mutations were verified by sequencing.

### Antibodies

MULAN polyclonal antibodies were generated by immunizing rabbits with a synthetic peptide corresponding to amino acids 57-76 (conserved between human and mouse), EAPGKCVPYAVIEGAVRSVK. The peptide antibody was further affinity-purified using an antigen column. Other primary antibodies used in this study were: Rabbit polyclonal antibodies against Ub (Z0458, DAKO, Carpinteria, CA), Tom20 (sc-11445, Santa Cruz Biotechnology, Santa Cruz, CA), Smac (Imgenex Corp., San Diego, CA), Sec61 (Dr. Nicholas Gekakis, TSRI) and Calreticulin (405-417, Calbiochem, San Diego, CA); Monoclonal antibodies against Golgin-97 (CDF4, Molecular Probes, Carlsbad, CA), the Flag tag (M2, Sigma), EEA1 (Transduction Laboratories, Lexington, KY), cytochrome-c (7H8.2C12, BD Pharmingen, La Jolla, CA) and alpha-tubulin (DM1A, Sigma). Secondary antibodies used were: Alexa Fluor 488 goat anti-rabbit and goat anti-mouse IgGs (Molecular Probes), Cy3 donkey anti-mouse IgG (Jackson ImmunoResearch Laboratories, Inc., West Grove, PA).

### Cell culture, transfection and cDNA library screening

HEK293, NIH3T3 and HeLa cells were maintained in DMEM supplemented with 10% FBS at 37°C in 5% CO2. DNA transfection was performed with lipofectamine 2000 (Invitrogen, Carsbad, CA) or Fugene 6 (Promega, Madison, WI). siRNAs were transfected using lipofectamine 2000.

The E3 cDNA library was arrayed in a 384-well plate format, ready for reverse transfection [17]. For the screen reported, each cDNA was co-transfected with MT-RFP. 24 h after transfection, cells were fixed in 4% formaldehyde and screened for cDNAs that led to obvious changes in mitochondrial morphology under the fluorescence microscope at 20× magnification.

siRNA oligos utilized were: siMULAN1, GAGCGCCUGUGUAGUGUGUUU (Dharmacon, Lafayette, CO); siMULAN2, CCGCGCCUUGCCAGAACCCAA (Qiagen, Valencia, CA); siScrambled (Qiagen # 1022076); and siCyclophilinB (Dharmacon). HeLa cells were transfected with siRNA on day 1 (at a final concentration of 50–100 nM in the culture media) followed by MT-RFP on day 2. Cells were harvested on day 3 either to collect RNA, or for fixation with 4% formaldehyde. Fixed cells were used to score for defective trafficking based on the perinuclear clustering of mitochondria, or were subjected to immunocytochemistry as described below.

### Immunocytochemistry and confocal microscopy

For immunocytochemistry, cells were plated out on 35-mm glass bottom dishes (MatTek Corp., Ashland, MA). Cells were fixed with 4% paraformaldehyde in phosphate-buffered saline (PBS) for 15 min, washed with PBS, permeabilized with 0.2% Triton X-100 for 5 min, washed four times with PBS and blocked with 3% bovine serum albumin, all at room temperature. Cells were then incubated with primary antibodies for 2 h at room temperature, washed three times with 0.2% Triton X-100, incubated with secondary antibodies for 30 min and washed again. Samples were mounted using Prolong Antifade (Invitrogen) and analyzed by confocal microscopy using an Olympus Fluoview 500 laser scanning confocal on an Olympus IX61 upright microscope. GFP was imaged with the 488-nm line of the Argon laser, and the emission filter was a 505–525 bandpass filter. RFP was imaged with 543 nm laser line from a HeNe green laser, and the emission filter was a 560–600 bandpass filter.

### Immuno-gold electron microscopy

Immuno-gold electron microscopy was performed at the University of California, San Diego, EM core. COS7 cells expressing MULAN-Flag were fixed in PBS containing 2% paraformaldehyde and 0.2% glutaraldehyde. Fixed cells were washed with 0.15 M glycine/PBS, embedded in 10% gelatin/PBS and infused overnight with 2.3 M sucrose/PBS at 4°C. 1-mm^3^ cell blocks were mounted onto specimen holders and snap frozen in liquid nitrogen. Ultracryomicrotomy was carried out at −100°C on a Leica Ultracut UCT with EM FCS cryoattachment (Leica, Bannockburn, IL) using a Diatome diamond knife (Diatome US, Fort Washington, PA). 60–70 nm frozen sections were picked up with a 1∶1 mixture of 2.3 M sucrose and 2% methyl cellulose and transferred onto Formvar and carbon-coated copper grids. Immunolabeling was performed by a slight modification of the “Tokuyasu technique.” Briefly, grids were placed on 2% gelatin at 37°C for 20 min, rinsed with 0.15% glycine/PBS and the sections were blocked using 1% cold water fish-skin gelatin. Primary antibody against Flag was diluted 1/100. Incubation with primary antibody for 1 h at room temperature was followed by 5-nm gold-conjugated goat anti-mouse IgG and IgM (RPN 430, Amersham Pharmacia Biotech, Piscataway, NJ) and 10-nm gold-conjugated goat anti-rabbit IgG (RPN 421), both diluted 1/25 in 1% BSA/PBS at room temperature for 30 min. Grids were viewed and photographed using a JEOL 1200EX II transmission electron microscope (JEOL, Peabody, MA).

### Gradient centrifugation, mitochondria isolation and trypsin digest

For gradient centrifugation, HEK293 cells expressing MULAN-Flag were harvested from a 10-cm dish, washed in PBS and collected by centrifugation at 600×g for 5 min. Cells were then washed with HE buffer (10 mM Hepes-KOH, pH 7.5, and 1 mM EDTA) containing 10% (wt/vol) sucrose. Cells were suspended in 1 ml of the HE buffer with 20 µg/ml α2-macroglobulin and protease inhibitor cocktail. Cells were passed through a 27 gauge needle five times for homogenization and centrifuged at 600×g for 10 min to obtain a post-nuclear supernatant. The supernatant was layered over a discontinuous gradient of 40% and 60% sucrose in HE buffer (6.6 and 2.2 ml, respectively). The gradient was centrifuged at 100,000×g for 3 h. 1ml aliquots were collected, 100 µl of each fraction was concentrated using microcon columns (Millipore, Billerica, MA) and subjected to SDS-PAGE. Immunoblotting was performed using anti-Golgin 97, anti-Tom20 and anti-Flag antibodies. Highly purified mitochondria were isolated using methods described by Rezaul and colleagues [30]. Cells were harvested by centrifugation at 1000 rpm for 5 min at room temperature, then resuspended in solution A (0.25 M sucrose, 20 mM HEPES-KOH, pH 7.5, 10 mM KCl, 1.5 mM MgCl_2_, 1 mM EDTA, 1 mM EGTA, 1 mM DTT) with protease inhibitor cocktail (Sigma). The cellular suspension was homogenized with a glass Dounce homogenizer with 40 times up and down passes of the pestle. The homogenate was centrifuged at 750×g for 15 min. The supernatant was centrifuged at 12,500×g for 25 min to collect crude mitochondria. The pellet was resuspended in solution B (0.25 M sucrose, 1 mM EDTA, 10 mM Tris-HCl pH 7.4) then layered on a 1 M/1.5 M discontinuous sucrose gradient and centrifuged at 60,000×g for 40 min. Mitochondria were collected from the interface and washed with at least 5 volumes of solution A for downstream applications. For trypsin digest, 50–100 µg of highly purified mitochondria were incubated with or without 25 µg/ml of trypsin protease in 50 µl total volume at 37°C for 5 sec, 5 min and 15 min, with or without 0.5% Triton-X 100. Reactions were stopped with trypsin inhibitor and run on SDS-PAGE to probe with anti-Flag, anti-Tom20 and anti-SMAC antibodies.

### *In vitro* ubiquitylation assay

In vitro ubiquitylation was performed as described [31], with His-Ubc4 and MULAN GST fusion proteins. GST fusion proteins were expressed and isolated as described [31].

### Gene expression analyses

RNA was isolated using the RNeasy kit (Qiagen). Semi quantitative RT-PCR was performed using the Superscript II reverse transcriptase (Invitrogen). TaqMan qRT-PCR was performed using the one-step Superscript III platinum reagent (Invitrogen). Samples were run in triplicate as multiplexed reactions with a normalizing internal control (36B4; probe and primer were gifts of Dr. E. Saez, TSRI). MULAN probe and primer were ordered from Applied Biosystems (Foster City, CA). Northern blot was performed with a commercial 12-lane multiple human tissue blot (Clontech) using full length human MULAN cDNA to generate the probe.

## Supporting Information

Figure S1 MULAN features. A) Hydrophobicity plot predicts two transmembrane domains in MULAN, amino acids 9-29 and 242-259. B) Alignment of the MULAN RNF from various species: human (*H. sapiens*), mouse (*M. musculus*), frog (*X. laevis*), fish (*D. rerio*), plant (*A. thaliana*), fly (*D. melanogaster*) and mosquito (*A. gambiae*). *, Zn-binding residues. Dark blue, evolutionarily conserved residues. C) Immuno-EM shows MULAN-GFP signal in mitochondria (M) but not in the Golgi (G) or nucleus (N), e.g. NIH3T3 cells transfected with MULAN-GFP were fixed and stained with anti-GFP antibody followed by gold-conjugated secondary antibody for immuno-EM analysis. Scale bar, 200nm. D) Perinuclear clustering of mitochondria dependent on the dose of ectopically expressed MULAN. *Bar graphs*: NIH3T3 cells were transfected with the empty vector, a serial dilution of plasmid encoding wild type MULAN cDNA, or the equivalent amount of MULAN 1-301 cDNA, together with MT-RFP. Cells were fixed at 24 h post-transfection and the percentage of MT-RFP positive cells was determined. *Western blot panels*: In experiments performed in parallel under the same conditions, whole cell extracts were used in blots with MULAN antibody to determine the relative levels of ectopically expressed protein for each plasmid dilution. Anti-tubulin blot was used as a loading control. E) MULAN does not colocalize with the Golgi marker, Lectin-Alexa 488. NIH3T3 cells were transfected with MULAN-Flag or untagged MULAN 1-301, followed by immunostaining with antibody against Flag (top) or MULAN (bottom), together with Lectin-Alexa 488 (green, Molecular Probes). F) NIH3T3 cells were transfected with MULAN-Flag, followed by immunostaining with antibody against Flag (red) and the ER marker, calreticulin (green). The apparent partial co-localization of MULAN with the ER is presumably an artifact of the widespread ER signal.
(6.16 MB TIF)

Figure S2 Expression of MULAN proteins. A) Peptide antibody against MULAN amino acids 57-76 was used to blot whole cell lysates of HEK293 cells transfected with MULAN wild type or mutant constructs. B) Anti-Flag tag antibody was used to blot whole cell lysates of HEK293 cells transfected with Flag-tagged MULAN wild type or mutant constructs. All constructs were Flag tagged at the C-terminus, except for the N-terminal tagged MULAN (lane 5). C) siRNA-mediated knockdown of MULAN protein. HeLa cells were transfected with the indicated siRNAs on day 1, MULAN cDNA on day 2. Cells were harvested 48 h after cDNA transfection. Whole cell lysates were blotted with anti-MULAN antibody. In all panels, anti-α-tubulin blot was used to control for protein loading.
(1.77 MB TIF)

Figure S3 Both MULAN ectopic expression and endogenous knockdown indicate a role in mitochondrial dynamics. For ectopic expression, HeLa cells were transfected with vector or MULAN wild type cDNA together with MT-RFP. Cells were fixed for analysis 24 h post-transfection. For siRNA-mediated knockdown, cells were transfected with siScrambled or siMULAN1 on day 1 and with MT-RFP on day 2. Cells were fixed on day 3. Fixed cells were immunostained with anti-α-tubulin antibody (green) to visualize the microtubule network. Mitochondria marked with MT-RFP are shown in red.
(7.74 MB TIF)

Figure S4 MULAN is targeted to mitochondria via signal-anchor type transmembrane domains (TMDs) and its optimal targeting requires multiple signals. NIH3T3 cells were transfected with the indicated GFP-fusion proteins, Flag-tagged point mutants or deletion constructs of MULAN. Localization of the proteins was revealed by GFP fluorescence or Flag immunostaining (green). Mitochondria were visualized using MT-RFP (red). A) MULAN lacks an N-terminal mitochondrial signal peptide. Upper: the N-terminal 33 amino acids of MULAN were not sufficient to target GFP to mitochondria. Lower: MULAN's N-terminal 10 amino acids were not required for mitochondrial localization. B) Isolated TMDs of MULAN combined with their flanking sequences were sufficient to target GFP to mitochondria. Upper: TMD1 targeted GFP to mitochondria when combined with a C-terminal stretch of basic amino acids (1-60). Lower: TMD2 together with additional N- and C-terminal sequences (amino acids 158-279) targeted GFP to mitochondria. C) Mutation of the basic residues immediately following TMD2 in the context of full-length MULAN-Flag (MULAN R260A/K261A) led to mislocalization to the ER. Upper: MULAN R260A/K261A does not colocalize with MT-RFP. Lower: MULAN R260A/K261A colocalizes with the ER marker, calreticulin. D) N-terminal Flag-tagged MULAN localized to cytosol. E) Deletion of the entire C-terminal cytoplasmic domain (MULAN 1-263) or of the RING domain (MULAN 1-301) did not affect MULAN's mitochondrial localization. (See also Text S1.)
(16.49 MB TIF)

Figure S5 MULAN sequences including TMD2 and immediately neighboring residues (amino acids 238-263) are not sufficient for targeting MULAN to mitochondria. NIH3T3 cells were transfected with a construct encoding the MULAN 238-263 fragment C-terminal tagged with GFP, alone or together with MT-RFP. Upper row: 238-263-GFP does not colocalize with the mitochondrial marker MT-RFP. Lower row: 238-263-GFP colocalizes with the Golgi marker, Golgin97.
(5.93 MB TIF)

Table S1 List of the predicted *H. sapiens* E3s.
(0.16 MB XLS)

Text S1 (0.05 MB DOC)
