# Supplementary figures and images for: Genome-Wide and Functional Annotation of Human E3 Ubiquitin Ligases Identifies MULAN, a Mitochondrial E3 that Regulates the Organelle's Dynamics and Signaling
Source: PLoS One. 2008 Jan 23;3(1):e1487. doi: 10.1371/journal.pone.0001487 (PMC2198940; doi:10.1371/journal.pone.0001487)

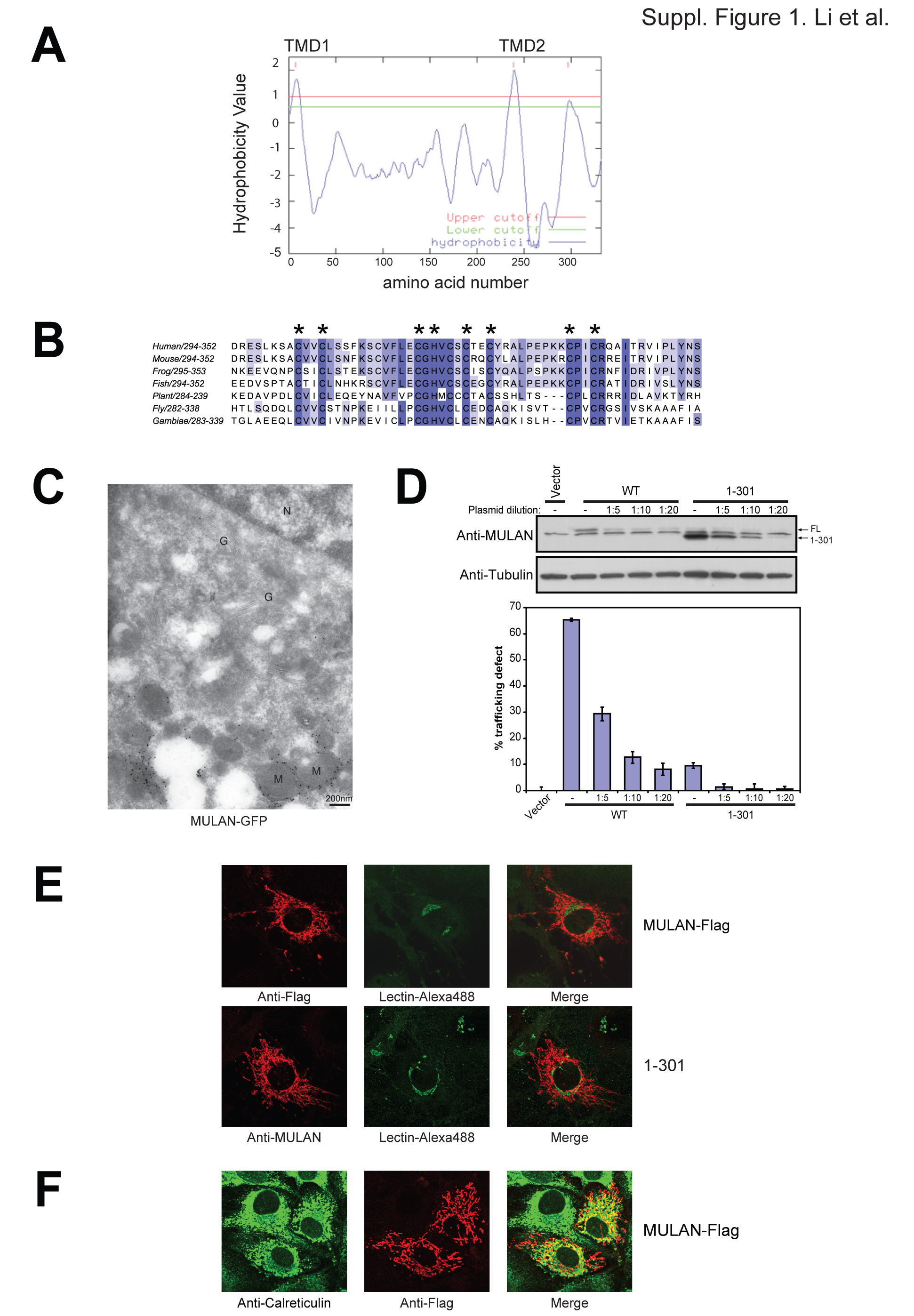

Supplement: Figure S1 — MULAN features. A) Hydrophobicity plot predicts two transmembrane domains in MULAN, amino acids 9-29 and 242-259. B) Alignment of the MULAN RNF from various species: human (H. sapiens), mouse (M. musculus), frog (X. laevis), fish (D. rerio), plant (A. thaliana), fly (D. melanogaster) and mosquito (A. gambiae). *, Zn-binding residues. Dark blue, evolutionarily conserved residues. C) Immuno-EM shows MULAN-GFP signal in mitochondria (M) but not in the Golgi (G) or nucleus (N), e.g. NIH3T3 cells transfected with MULAN-GFP were fixed and stained with anti-GFP antibody followed by gold-conjugated secondary antibody for immuno-EM analysis. Scale bar, 200nm. D) Perinuclear clustering of mitochondria dependent on the dose of ectopically expressed MULAN. Bar graphs: NIH3T3 cells were transfected with the empty vector, a serial dilution of plasmid encoding wild type MULAN cDNA, or the equivalent amount of MULAN 1-301 cDNA, together with MT-RFP. Cells were fixed at 24 h post-transfection and the percentage of MT-RFP positive cells was determined. Western blot panels: In experiments performed in parallel under the same conditions, whole cell extracts were used in blots with MULAN antibody to determine the relative levels of ectopically expressed protein for each plasmid dilution. Anti-tubulin blot was used as a loading control. E) MULAN does not colocalize with the Golgi marker, Lectin-Alexa 488. NIH3T3 cells were transfected with MULAN-Flag or untagged MULAN 1-301, followed by immunostaining with antibody against Flag (top) or MULAN (bottom), together with Lectin-Alexa 488 (green, Molecular Probes). F) NIH3T3 cells were transfected with MULAN-Flag, followed by immunostaining with antibody against Flag (red) and the ER marker, calreticulin (green). The apparent partial co-localization of MULAN with the ER is presumably an artifact of the widespread ER signal. (6.16 MB TIF) [file pone.0001487.s001.tif]

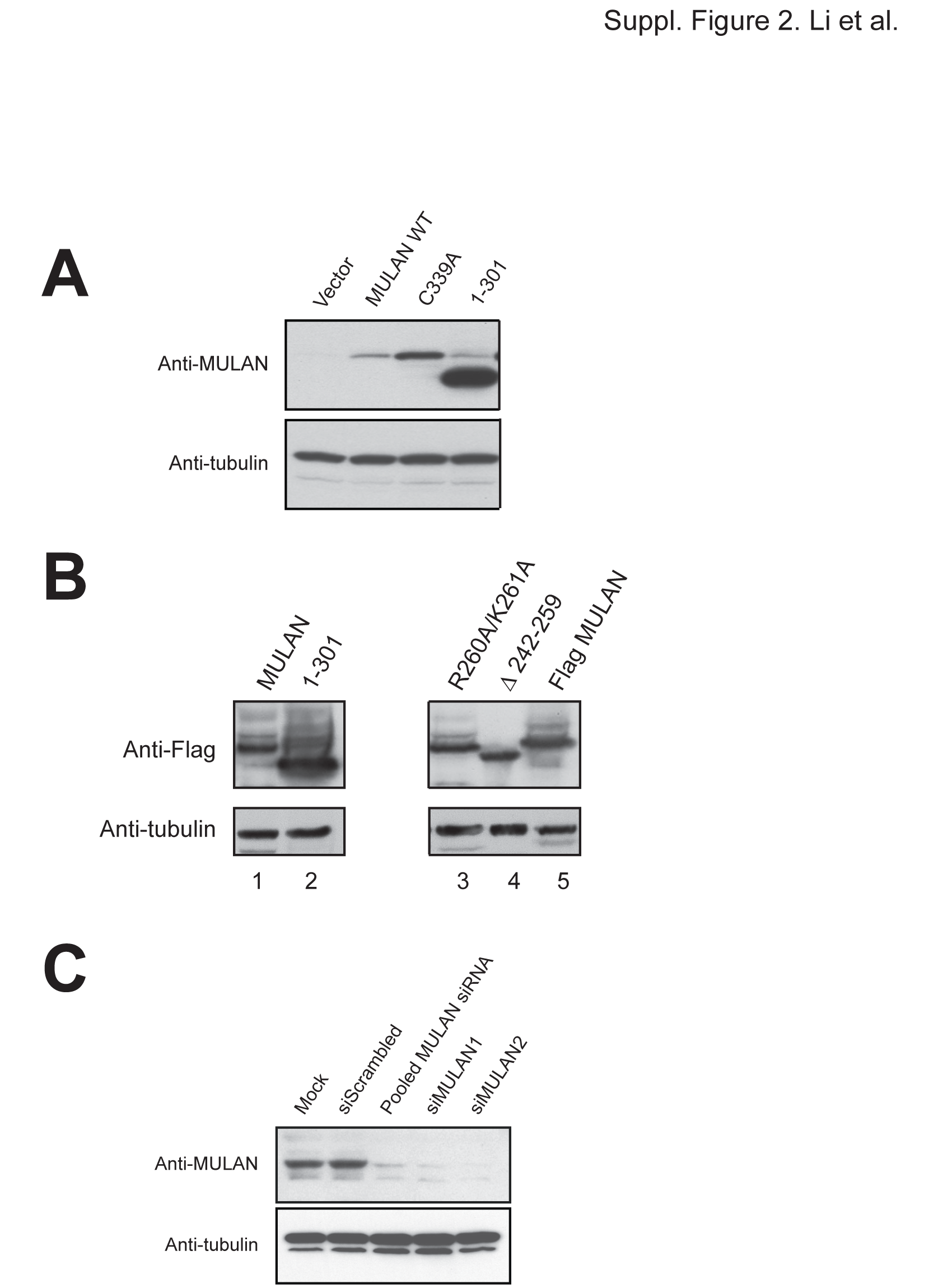

Supplement: Figure S2 — Expression of MULAN proteins. A) Peptide antibody against MULAN amino acids 57-76 was used to blot whole cell lysates of HEK293 cells transfected with MULAN wild type or mutant constructs. B) Anti-Flag tag antibody was used to blot whole cell lysates of HEK293 cells transfected with Flag-tagged MULAN wild type or mutant constructs. All constructs were Flag tagged at the C-terminus, except for the N-terminal tagged MULAN (lane 5). C) siRNA-mediated knockdown of MULAN protein. HeLa cells were transfected with the indicated siRNAs on day 1, MULAN cDNA on day 2. Cells were harvested 48 h after cDNA transfection. Whole cell lysates were blotted with anti-MULAN antibody. In all panels, anti-α-tubulin blot was used to control for protein loading. (1.77 MB TIF) [file pone.0001487.s002.tif]

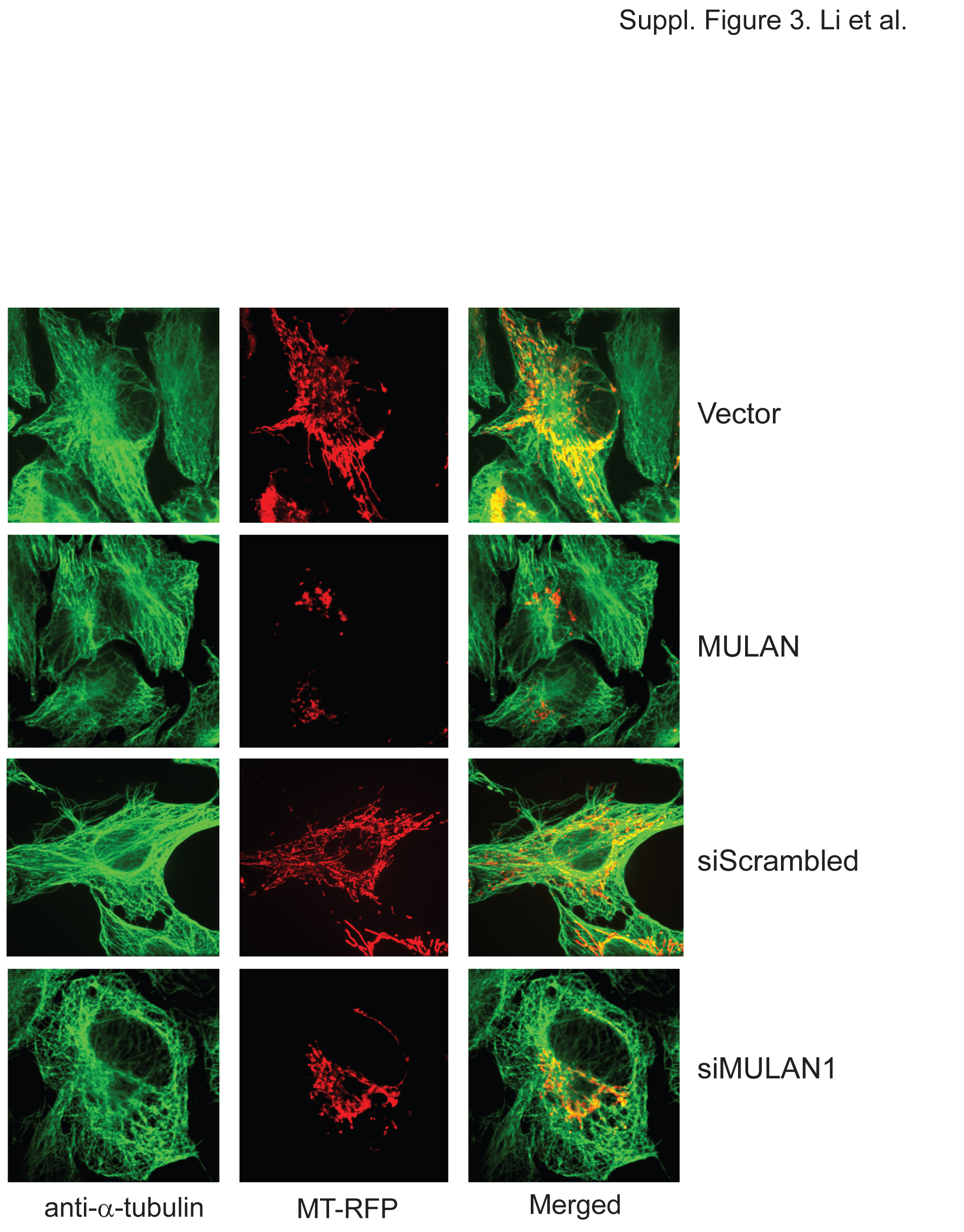

Supplement: Figure S3 — Both MULAN ectopic expression and endogenous knockdown indicate a role in mitochondrial dynamics. For ectopic expression, HeLa cells were transfected with vector or MULAN wild type cDNA together with MT-RFP. Cells were fixed for analysis 24 h post-transfection. For siRNA-mediated knockdown, cells were transfected with siScrambled or siMULAN1 on day 1 and with MT-RFP on day 2. Cells were fixed on day 3. Fixed cells were immunostained with anti-α-tubulin antibody (green) to visualize the microtubule network. Mitochondria marked with MT-RFP are shown in red. (7.74 MB TIF) [file pone.0001487.s003.tif]

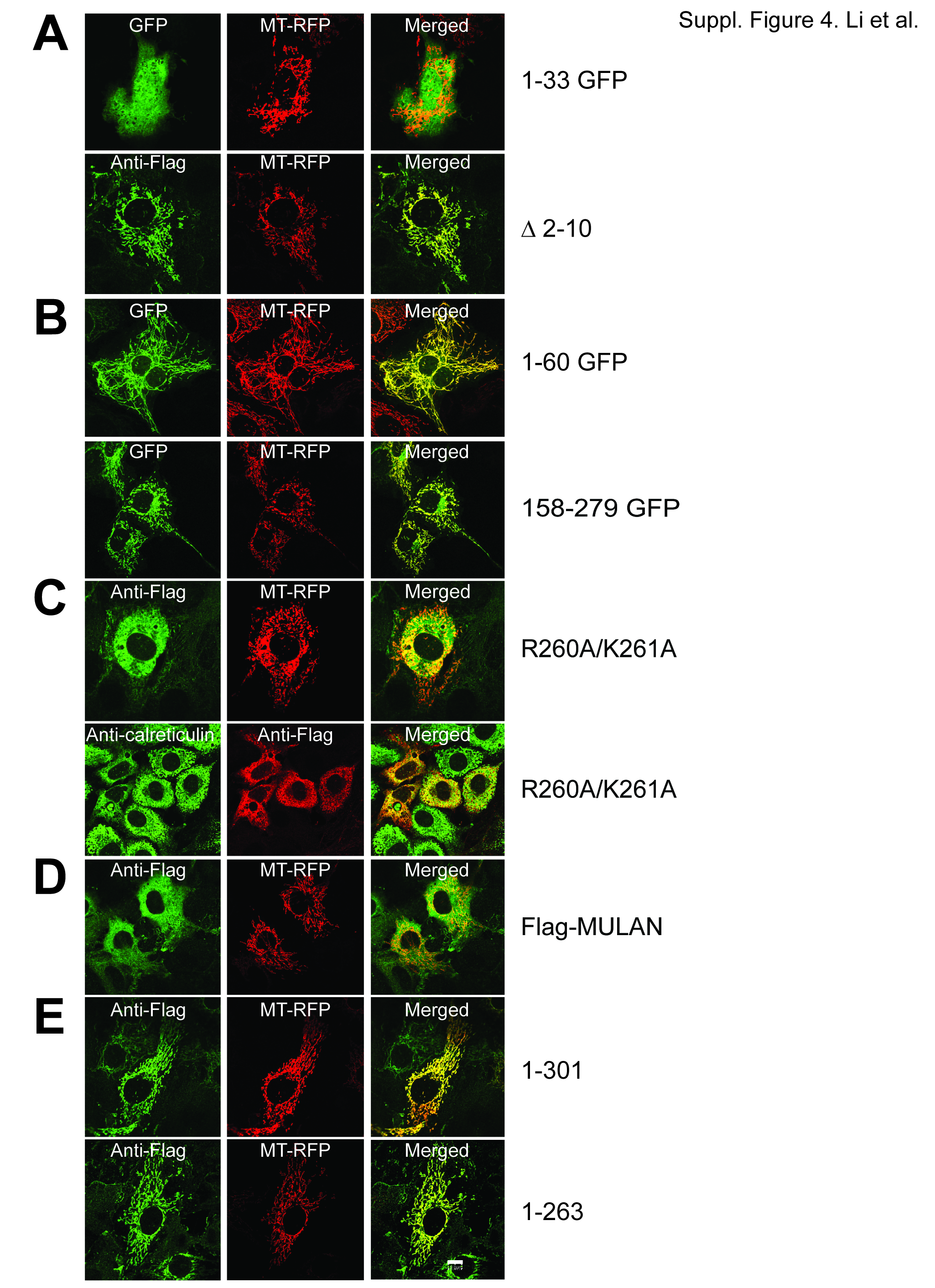

Supplement: Figure S4 — MULAN is targeted to mitochondria via signal-anchor type transmembrane domains (TMDs) and its optimal targeting requires multiple signals. NIH3T3 cells were transfected with the indicated GFP-fusion proteins, Flag-tagged point mutants or deletion constructs of MULAN. Localization of the proteins was revealed by GFP fluorescence or Flag immunostaining (green). Mitochondria were visualized using MT-RFP (red). A) MULAN lacks an N-terminal mitochondrial signal peptide. Upper: the N-terminal 33 amino acids of MULAN were not sufficient to target GFP to mitochondria. Lower: MULAN's N-terminal 10 amino acids were not required for mitochondrial localization. B) Isolated TMDs of MULAN combined with their flanking sequences were sufficient to target GFP to mitochondria. Upper: TMD1 targeted GFP to mitochondria when combined with a C-terminal stretch of basic amino acids (1-60). Lower: TMD2 together with additional N- and C-terminal sequences (amino acids 158-279) targeted GFP to mitochondria. C) Mutation of the basic residues immediately following TMD2 in the context of full-length MULAN-Flag (MULAN R260A/K261A) led to mislocalization to the ER. Upper: MULAN R260A/K261A does not colocalize with MT-RFP. Lower: MULAN R260A/K261A colocalizes with the ER marker, calreticulin. D) N-terminal Flag-tagged MULAN localized to cytosol. E) Deletion of the entire C-terminal cytoplasmic domain (MULAN 1-263) or of the RING domain (MULAN 1-301) did not affect MULAN's mitochondrial localization. (See also Text S1.) (16.49 MB TIF) [file pone.0001487.s004.tif]

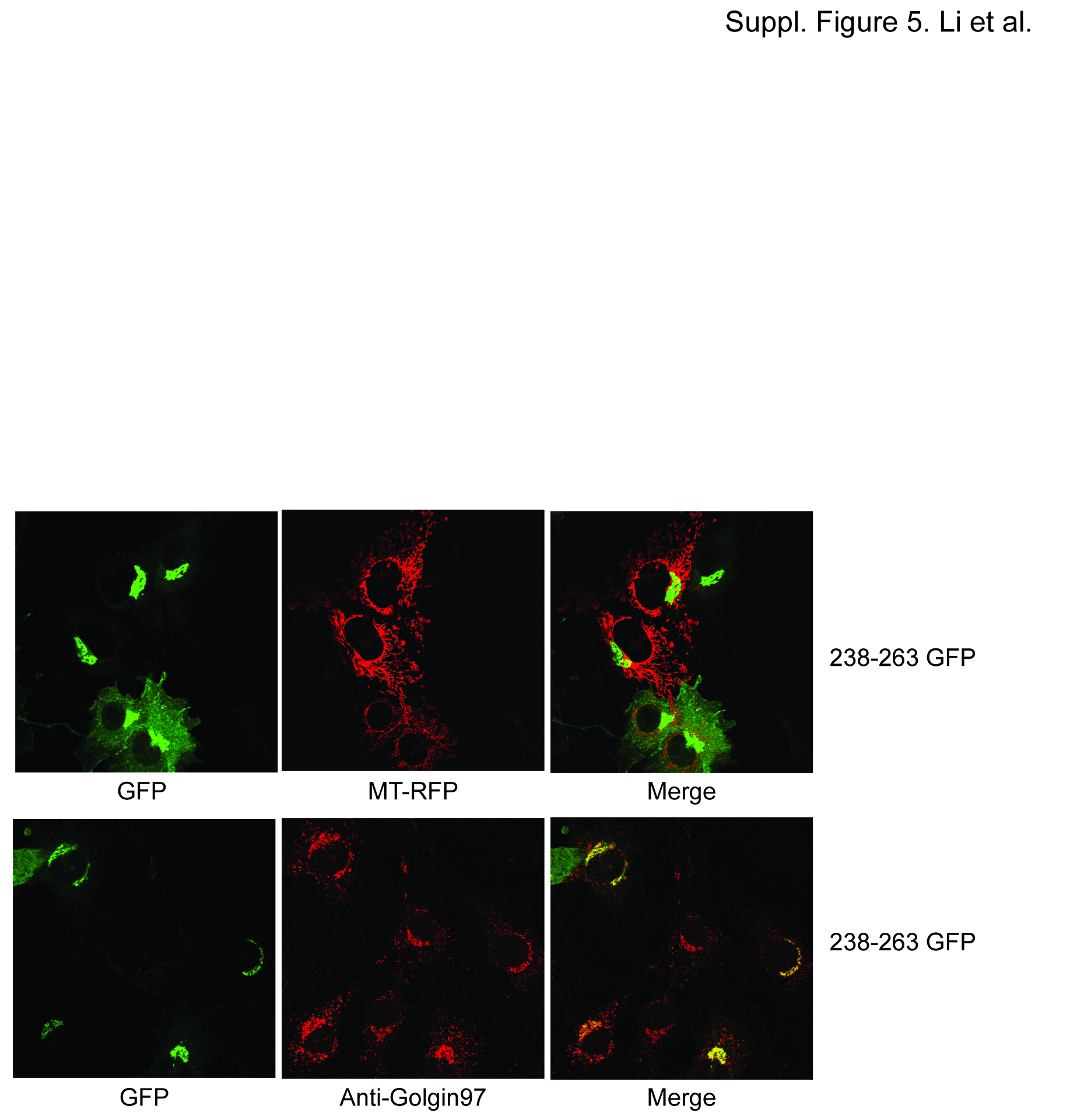

Supplement: Figure S5 — MULAN sequences including TMD2 and immediately neighboring residues (amino acids 238-263) are not sufficient for targeting MULAN to mitochondria. NIH3T3 cells were transfected with a construct encoding the MULAN 238-263 fragment C-terminal tagged with GFP, alone or together with MT-RFP. Upper row: 238-263-GFP does not colocalize with the mitochondrial marker MT-RFP. Lower row: 238-263-GFP colocalizes with the Golgi marker, Golgin97. (5.93 MB TIF) [file pone.0001487.s005.tif]
