# Supplementary material for: Genome-Wide and Functional Annotation of Human E3 Ubiquitin Ligases Identifies MULAN, a Mitochondrial E3 that Regulates the Organelle's Dynamics and Signaling
Source: PLoS One. 2008 Jan 23;3(1):e1487. doi: 10.1371/journal.pone.0001487 (PMC2198940; doi:10.1371/journal.pone.0001487)
Supplement: Text S1 — (0.05 MB DOC) [file pone.0001487.s007.doc]

**Text S1**

For many nuclear-encoded MOM proteins, transmembrane sequences with moderate hydrophobicity and net positive charge in flanking regions serve as the *required* and *sufficient* signals for post-translational targeting, known as signal-anchor or TMD+ domains [1]. This is in contrast with the cleavable N-terminal sequence that normally targets proteins to the mitochondrial inner membrane and matrix [2]. To further define the structural elements implicated in MULAN’s mitochondrial localization, we generated this series of deletion constructs, point mutations and N- or C-terminally tagged proteins. These were expressed in NIH3T3 cells and their subcellular localization was determined by immunocytochemistry. Representative results are presented in **Figure S4**.

1. Role of the N-terminus. Unlike for the 25 N-terminal amino acids of cytochrome c oxidase subunit VIII, fusion of MULAN’s N-terminal 33 amino acids to GFP were not sufficient for mitochondrial targeting (panels A, upper row); conversely, MULAN’s mitochondrial localization was unaffected by deletion of its N-terminal 10 amino acids (panels A, lower row). However, placing a Flag tag at MULAN’s N-terminus led to the protein’s mislocalization to the cytosol (panels D).

2. Role of TMDs. Each isolated TMD sufficed for mitochondrial targeting provided that additional flanking sequences were present (panels B). For example, a construct encoding TMD2 and adjacent residues, including the two basic residues at its C-terminus (238-263 GFP) largely misdirected the protein to the Golgi apparatus (see Fig.S5). Only when a C-terminal fragment enriched for basic residues and N-terminal flanking sequences were both included (amino acids 158-279), did the fusion protein correctly localize to mitochondria (panels B, lower row); strikingly, mutation of the two positively charged residues C-terminal to TMD2 in the context of the full-length protein (R260A/K261A) led to its mistargeting (panels C, upper row). Since analogous mutations had already been shown to mistarget the MOM proteins Tom20 and Mfn2 to the endoplasmic reticulum (ER) [3, 4], we tested whether the same was true for MULAN R260A/K261A. In some cells, C-terminal Flag-tagged R260A/K261A largely colocalized with the ER marker, calreticulin (panels C, lower row); in other cells, it showed mixed localization to cytoplasm, mitochondria and ER (unpublished observations).

3. Role of the C-terminus. Deletion of either the RNF (mutant 1-301) or of cytoplasmic sequences altogether (mutant 1-263) failed to affect MULAN’s mitochondrial localization (panels E), suggesting the absence of an essential targeting signal at the C-terminus.

The effect of the N-terminal Flag tag in promoting the protein’s mislocalization to the cytosol was unexpected, given that MULAN’s N-terminus is neither required nor sufficient for mitochondrial targeting (panel A) and that TMD+ domains in other proteins can act at various distances from the N-terminus [1]. This effect was not specific to the Flag tag, since N-terminally GFP-tagged MULAN was likewise localized to the cytoplasm (unpublished observations). We conclude that MULAN’s targeting to mitochondria is mediated by the two TMD+ domains, similarly to what has been described for Mfn2 [4, 5], and that it can be influenced by the N-terminus.
